# The Effects of Group Integrative Arts Therapy based on Social Skill Training on the Social Adaptive Function, Empowerment and Subjective Well-Being in Inpatients with Chronic Schizophrenia

**DOI:** 10.1192/j.eurpsy.2025.1605

**Published:** 2025-08-26

**Authors:** E. Lim, S.-Y. Lee

**Affiliations:** 1 Psychiatry, Shinsegae Hyo Hospital, Gimje; 2 Psychiatry, Wonkwang University, School of medicine, Iksan, Korea, Republic Of

## Abstract

**Introduction:**

The social skills of the chronic schizophrenia is an important factor in assessing the prognosis of patients with schizophrenia.

**Objectives:**

The object of this study was to investigate the effects of group integrative arts therapy based on social skill training on communication, social adaptive function, and subjective well-being in inpatients with chronic schizophrenia.

**Methods:**

Among the 125 patients who had been hospitalized in the mental hospital after being diagnosed with schizophrenia by psychiatrists according to DSM-5, 72 patients were selected by inclusion criteria and 48 patients were randomly assigned into an experimental group(n=16), comparative group(n=16), and control group(n=16). During this study, 4 patients from each groups dropped out. The final subjects of each groups were 12 patients. The experimental group followed a 60 minutes long social skill training based on group integrative arts therapy program for twice a week and 20 times in total. The Comparative group followed a social skill training program only for 60 minutes twice a week for 20 times in total. The control group received no treatment. To assess the social adaptive function, empowerment, subjective well-being of the subjects, Communication Competence Scale(CCS), Empowerment Scale(ES) and Korean Modification of Subjective Well-Being Scale(KmSWN) were used as subjective measuring. Assertiveness Observation Evaluation Scale(AOES), Social Adaptive Functioning Scale(SAFS), and Nurses’ Observation Scale of Inpatient Evaluation-30(NOSIE-30) were also used as objective measuring that were rated by nurses or social workers at the mental hospital.

**Results:**

There was no statistically significant difference except non-verbal communication of CCS among three groups in homogeneity test of sociodemographic and clinical variables. The group integrative arts therapy based on social skill training was found to significantly increase the communication, assertiveness, social adaptive functioning and empowerment of experimental group more than comparative group, and that of comparative group more than the control group. The group integrative arts therapy based on social skill training was found to significantly increase the NOSIE-30 of the experimental group and the comparative group more than control group. NOSIE-positive and irritability of NOSIE-30 in the comparative group was increased more than those of the experimental and the control groups.

**Conclusions:**

The group integrative arts therapy based on social skill training is found to significantly enhance the social adaptive function and empowerment of inpatients with chronic schizophrenia than social skill training. These results suggest that group integrative arts therapy could be utilized as effective mental rehabilitation intervention program for inpatients with chronic schizophrenia.

**Disclosure of Interest:**

None Declared

